# Multicenter validation of the newly developed Concise Objectifiable Risk Evaluation (CORE) score also confirms its ability to complement the Hematopoietic Cell Comorbidity Index (HCT-CI)

**DOI:** 10.1038/s41409-025-02778-w

**Published:** 2025-12-16

**Authors:** Gunnar Weise, Christina Rautenberg, Alexander Denk, Radwan Massoud, Elisabeth Meedt, Daniel Wolff, Thomas Schroeder, Francis Ayuk

**Affiliations:** 1 Department of Stem Cell Transplantation, University Medical Center Hamburg-Eppendorf, Hamburg, Germany; 2 Department of Hematology and Stem Cell Transplantation, University Hospital Essen, Essen, Germany; 3 Department of Internal Medicine III, University Hospital Regensburg, Regensburg, Germany

**Keywords:** Haematological cancer, Haematopoietic stem cells, Cancer therapy

## Introduction

Allogeneic hematopoietic stem cell transplantation (allo-HCT) is a potentially curative treatment option for patients with various hematologic malignancies. Over the past decades, advancements in conditioning regimens, post-transplant care and supportive therapies have extended the availability of allo-HCT to elderly and/or frail patient populations. Reduced-intensity conditioning (RIC) and myeloablative conditioning (MAC) have been optimized to improve outcomes in these patients by reducing transplant-related mortality (TRM) while maintaining anti-malignancy effects [1–3]. The success of allo-HCT is dependent on appropriate patient selection, which requires the development of objective risk stratification tools to identify patients at risk of severe complications and mortality. Identifying high-risk patients may allow for improvement of amendable risk factors, individualized treatment approaches such as selecting alternative conditioning regimens or choosing alternative non-transplant treatment approaches [4].

Traditional tools such as the Hematopoietic Cell Transplantation Comorbidity Index (HCT-CI) have been widely used to assess transplant risk [5, 6]. However, other models have sought to incorporate more refined, objective parameters to enhance reproducibility across centers and predictive accuracy for organ systems at risk for complications [7–11].

The Concise Objectifiable Risk Evaluation (CORE) score incorporates laboratory and functional organ parameters: serum albumin, serum creatinine, C-reactive protein (CRP), and cardiopulmonary function parameters, allowing for a more objectively quantifiable and reproducible assessment. The CORE score was derived from a large single-center cohort and internally validated at the same institution [12]. This retrospective study sought to externally validate the CORE score.

## Patients and Methods

### Population Characteristics

A total of 446 patients from two external centers (Essen *N* = 387 and Regensburg *N* = 59) with a median age of 58 years (interquartile range [IQR], 47–64), who had undergone allo-HCT between 2020 and 2022 for acute myeloid leukemia (AML, 51%), acute lymphoblastic leukemia (ALL, 12%), myelodysplastic syndrome and myeloproliferative neoplasms (MDS/MPN, 28%), Multiple Myeloma (1.3%) or lymphomas (7%) were analyzed. HLA-matched unrelated donors were used in 71%, followed by HLA-matched related donors (16%). Haplo-identical related and other HLA-mismatched related donors were used in 9.5% of cases. Myeloablative conditioning was performed in 79%, while reduced intensity conditioning was used for the remaining 21% of patients (Supplementary Table 1).

### Statistical analysis

Analysis included a total of 384 patients for whom full data were available. Univariable and multivariable analysis (MVA) for cumulative incidence of NRM (with relapse as competing risk) included transplant-related parameters (conditioning intensity, donor gender, recipient gender, donor type, stem cell source, Karnofsky index, and underlying disease) and CORE score risk groups. A comparison between CORE score and HCT-CI was conducted using the area under the receiver operating characteristic (ROC) curve, and the findings are presented as c-statistics with the corresponding standard error (SE). All reported *p*-values are two-sided, and *p*-values < 0.05 were considered statistically significant. Statistical analysis was performed using SPSS (version 27) and RStudio (version 2024.04.1).

## Results

### Univariate analysis

**Fig. 1:**
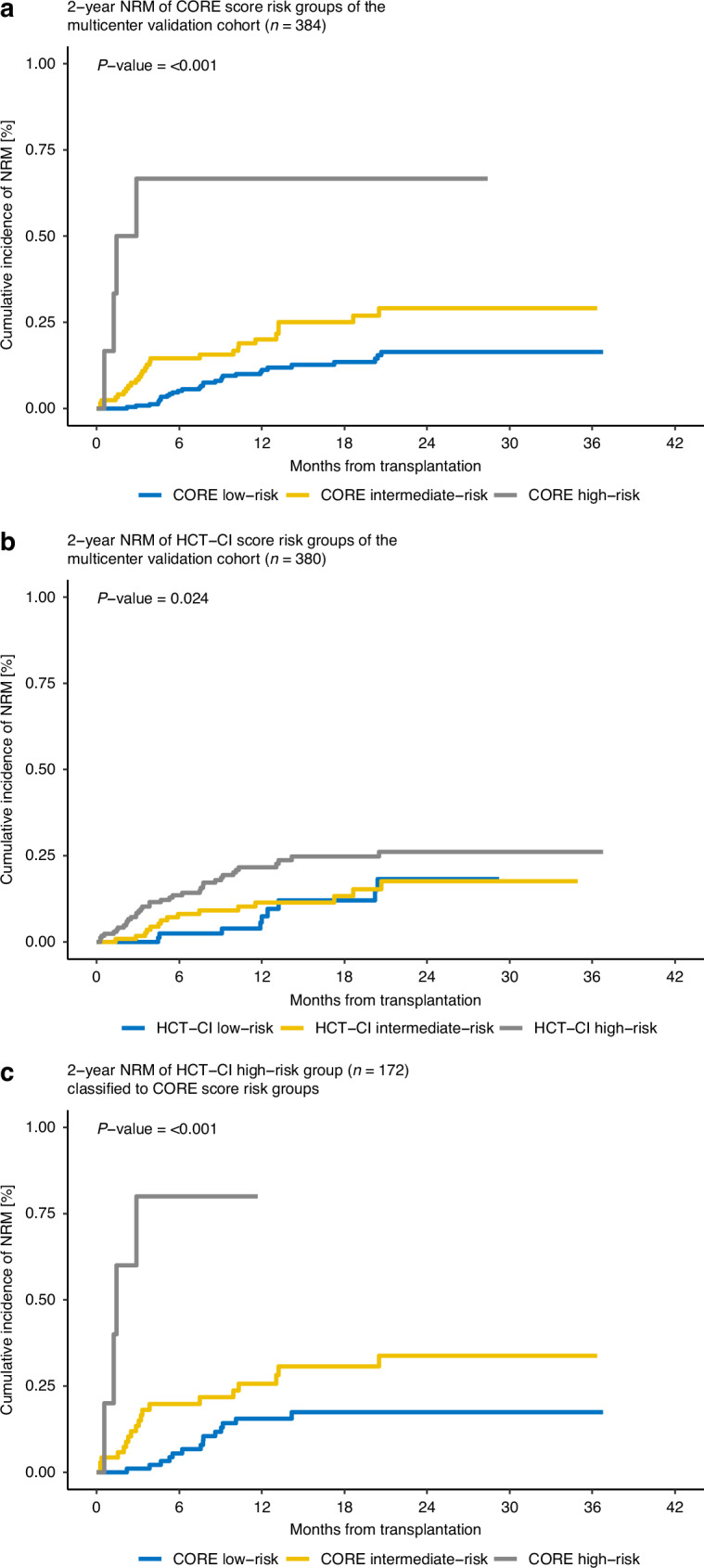
2-year non-relapse mortality (NRM) rates for the multicenter validation cohort. **a** 2-year NRM of CORE score for 384 patients. **b** 2-year NRM of HCT-CI score for 380 patients. HCT-CI was unable to separate three risk groups; **c** CORE score identifies a distinct low- and intermediate-risk group within 172 high-risk patients classified by HCT-CI.

Among 384 patients with a median follow-up for NRM of 349 days, low-risk and intermediate-risk groups accounted for 65% and 33% of the patients with 30 and 27 observed events, respectively. Only a very small population of 4 patients (1.5%) were high-risk. The corresponding NRM rates 2 years after allo-HCT were 16% for the low-risk group, 29% for the intermediate-risk group, and 67% for the high-risk group (*p* < 0.001). OS rates at 2 years posttransplant were 70%, 55% and 33%, respectively, with statistically significant differences among the three groups (*p* < 0.001). No significant results could be observed with regard to the relapse rates (*p* = 0.466), (Fig. 1a, Supplementary Fig. 2, Supplementary Tables 2, 3).

### Multivariate analysis

Only the CORE score was significantly associated with NRM in the univariate analysis. For MVA, relevant transplant-related parameters (disease entity, conditioning intensity, donor and recipient gender, donor type, stem cell source, and Karnofsky index) were, however, included to account for possible confounders. In the MVA, only the CORE score and underlying disease (MDS/MPN compared to AML) were significantly associated with NRM (Supplementary Tables 2, 3).

### Comparison of CORE score and HCT-CI

The HCT-CI has a high proportion of high-risk patients, with 28% in the original Seattle population [5]. In our analysis, 172 of 380 (45%) patients were identified as high-risk by HCT-CI with a 2-year-NRM of 26% (Fig. 1b). More than half of these patients (*n* = 96) are classified to the lowest CORE risk group and 71 patients are classified to the intermediate-risk group (Supplementary Fig. 3). The respective 2-year-NRM of patients with HCT-CI high-risk classified to the low-risk group by CORE was 16% compared to 29% for those who were classified to CORE intermediate-risk group (Fig. 1c). Using receiver operator characteristics (ROC) analysis, we compared CORE score with the HCT-CI. The respective c-statistics of 0.605 compared to 0.598 indicate that the CORE score performed at least as well as HCT-CI despite including only seven variables (Supplementary Table 4).

## Discussion

The ability of the HCT-CI to clearly separate patients into three distinct risk groups does not seem to hold in recently treated patients (Fig. 1b). When classified using the CORE model, the HCT-CI high-risk group is more than halved (172 down to 96 patients, minus 56%) and can therefore be separated into distinct low- and intermediate-risk groups (Fig. 1c). Transplantation could therefore be withheld from a group of patients because they are classified in the HCT-CI high-risk category by accumulating three relatively low-risk comorbidities. The CORE score can help identify patients within the high-risk group who do not exhibit a particularly high NRM by its ability to detect a very distinct high-risk patient population, characterized by poor organ function.

In direct comparison to the HCT-CI, the CORE score displayed robust performance while improving simplicity and objectivity. Notably, the HCT-CI is based on data collected more than two decades ago, whereas the CORE score is based on patient data from the past decade, reflecting advances in transplant techniques and supportive care strategies.

An important yet unanswered question is whether the improvement of modifiable risk factors e.g., as included in the CORE score, will lead to improved patient outcomes. If this is the case, pre-transplant interventions aiming to improve detected risk factors will become an essential part of transplant strategies. The parameters included in the CORE score may all be targets of pre-transplant interventions.

## Limitations

While results were similar across the original and validation analyses, a significant constraint lies in the relatively small size of the validation cohort, which impacted the MVA, where missing data led to an even smaller evaluable population. Regional or institutional differences may further contribute to the underrepresentation of high-risk patients. Consequently, only four patients were classified as high-risk, and overall confidence intervals remained relatively broad. The extremely high NRM of 67% observed within the high-risk group may at least in part be associated with the small number of high-risk patients.

## Conclusion

In summary, the univariate, multivariate, and comparative analyses of the CORE score reveal a tool with considerable strengths. Its objectivity and simplicity position it as a promising instrument for allo-HCT risk evaluation. However, the relatively small high-risk population observed in the external validation cohort underscores the need for further external validations and potential refinements. In conclusion, multicenter data further validate the predictive power of the CORE score and its robustness across diverse clinical settings.

## Supplementary information

Supplementary Figure 2: 2-year OS for CORE score of the multicenter validation cohort (n=384)

Supplementary Figure 3: Sankey plot with reclassification of HCT-CI risk score to CORE risk score

Supplementary Figure 4: 2-year OS for HCT-CI risk groups of the multicenter validation cohort (n=395)

Supplementary Table 1: Population characteristics

Supplementary Table 2: Prognostic factors for non-relapse mortality

Supplementary Table 3: External validation: CORE risk score prediction for non-relapse mortality, overall survival and relapse

Supplementary Table 4: ROC analysis for non-relapse mortality for CORE score and HCT-CI

Supplementary Table 5: Univariate analysis

## Data Availability

The datasets generated during and analyzed during the current study are not publicly available due to data privacy protection, but are available from the corresponding author on reasonable request.
