# Supplementary figures and images for: Multicenter validation of the newly developed Concise Objectifiable Risk Evaluation (CORE) score also confirms its ability to complement the Hematopoietic Cell Comorbidity Index (HCT-CI)
Source: Bone Marrow Transplant. 2025 Dec 16;61(3):322–5. doi: 10.1038/s41409-025-02778-w (PMC12965864; doi:10.1038/s41409-025-02778-w)

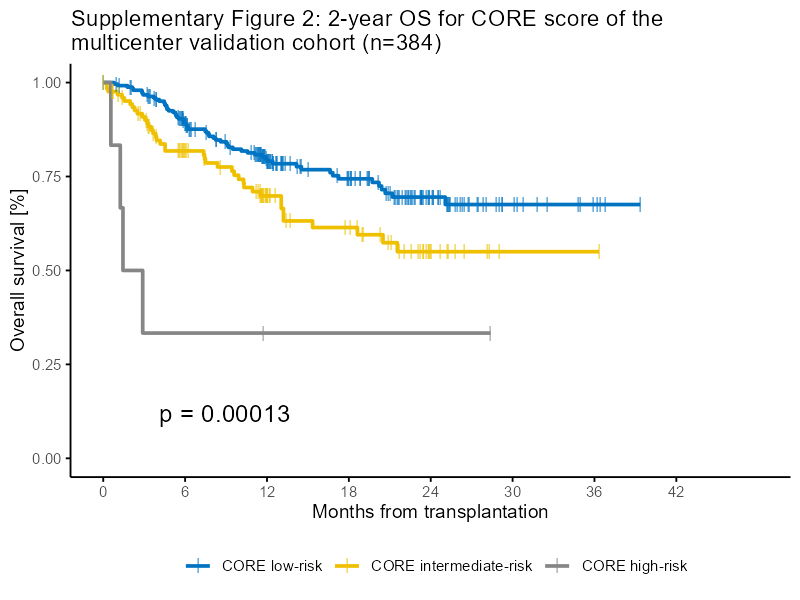

Supplement: Supplementary file 1 — Supplementary Figure 2: 2-year OS for CORE score of the multicenter validation cohort (n=384) [file 41409_2025_2778_MOESM1_ESM.png]

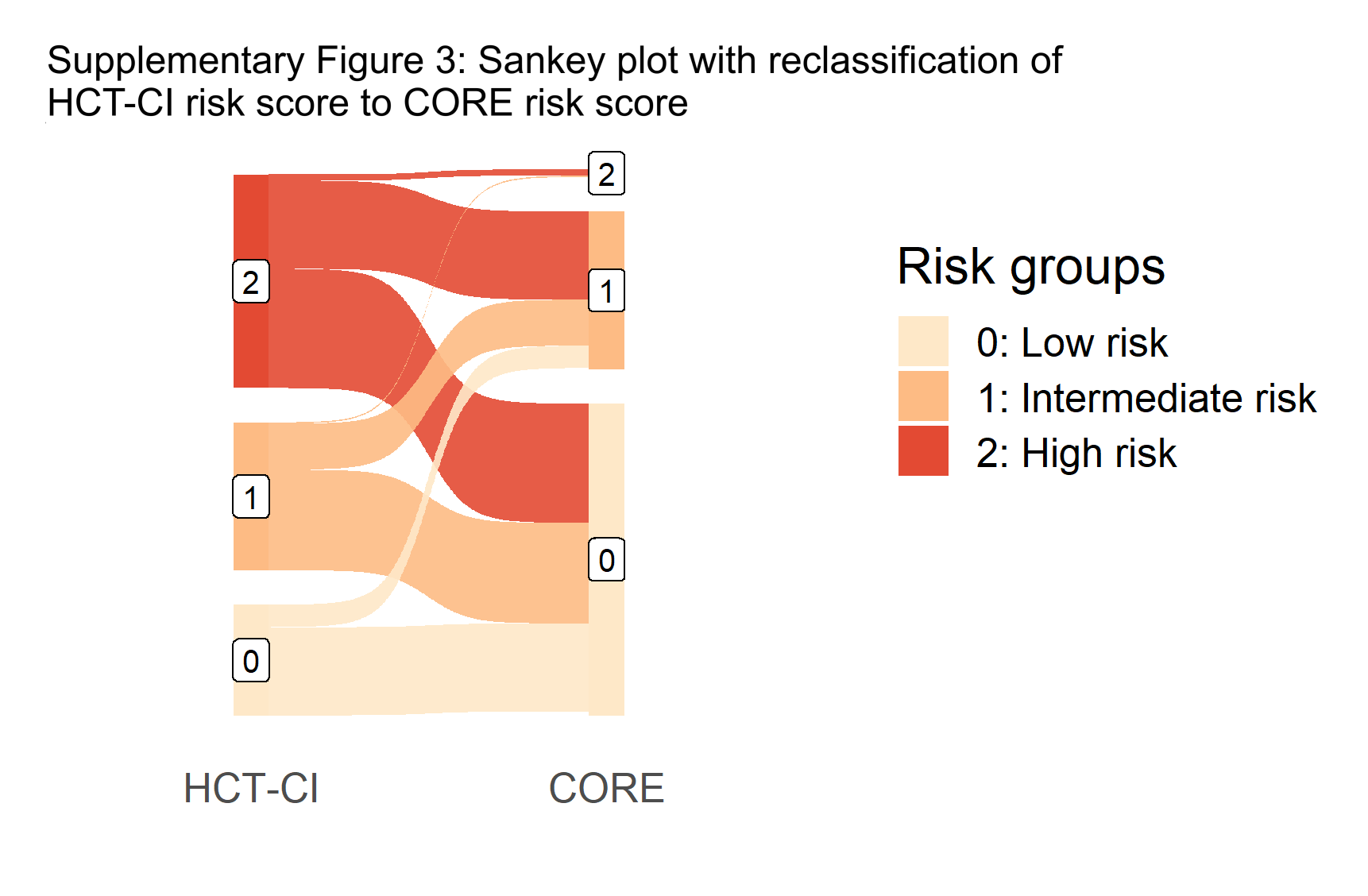

Supplement: Supplementary file 2 — Supplementary Figure 3: Sankey plot with reclassification of HCT-CI risk score to CORE risk score [file 41409_2025_2778_MOESM2_ESM.png]

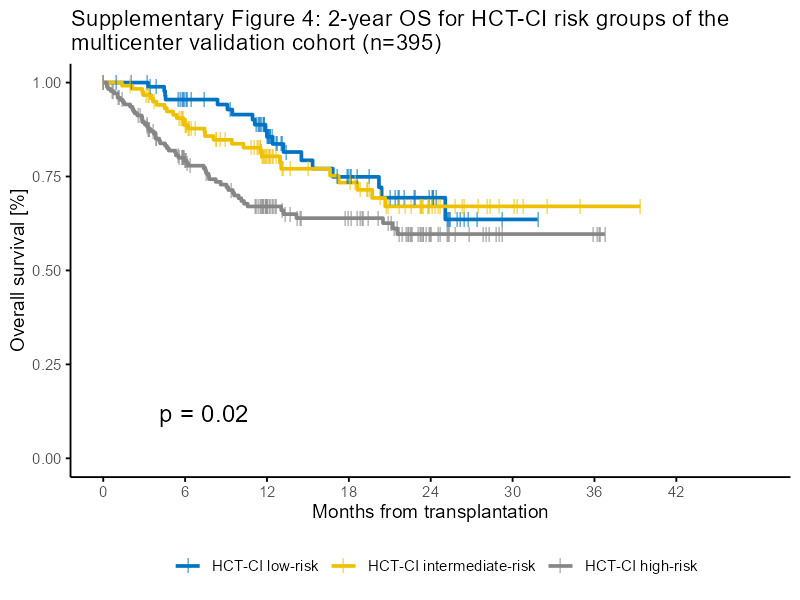

Supplement: Supplementary file 3 — Supplementary Figure 4: 2-year OS for HCT-CI risk groups of the multicenter validation cohort (n=395) [file 41409_2025_2778_MOESM3_ESM.png]
